# Melting dynamics of short dsDNA chains in saline solutions

**DOI:** 10.1186/s40064-015-1581-7

**Published:** 2015-12-15

**Authors:** Yichen He, Yazhuo Shang, Yu Liu, Shuangliang Zhao, Honglai Liu

**Affiliations:** State Key Laboratory of Chemical Engineering and Department of Chemistry, East China University of Science and Technology, Shanghai, 200237 China; State Key Laboratory of Chemical Engineering, East China University of Science and Technology, Shanghai, 200237 China

**Keywords:** DNA, Melting dynamics, Molecular simulation, Ionic concentration

## Abstract

DNA melting has attracted much attention due to its importance in understanding the life-reproduction and metabolism and in the applications of modern DNA-based technologies. While numerous works have been contributed to the determination of melting profiles in diverse environments, the understanding of DNA melting dynamics is still limited. By employing three-site-per-nucleotide (3SPN) double-stranded DNA (dsDNA) model, we here demonstrate the melting dynamics of an isolated short dsDNA under different conditions (different temperatures, ionic concentrations and DNA chain lengths) can be accessed by coarse-grained simulation studies. We particularly show that at dilute ionic concentration the dsDNA, regardless being symmetric or asymmetric, opens at both ends with roughly equal probabilities, while at high ionic concentration the asymmetric dsDNA chain opens at the A-T-rich end. The comparisons of our simulation results to available data are discussed, and overall good agreements have been found.

## Background

Denaturation of duplex DNA by heat leading to strand separation is known as DNA melting, and such phenomenon has caused more and more research interests in recent decades due to its importance in understanding the life-reproduction and metabolism. Primarily, a double-stranded DNA (dsDNA) dissociating into two single-stranded DNAs (ssDNA) lays a cornerstone for transcribing genetic information from DNA to RNA (Wang et al. 1998; Rief and Spudich 1999), and this process is indispensable during the replication and transcription of DNA. The DNA denaturation is also utilized in developing modern DNA-based technologies (Mirkin et al. 1996; Jin et al. 2003; Wu et al. 2011; Belozerova and Levicky 2012; Song et al. 2013; Wu et al. 2013). For instances, Jin et al. (2003) showed that the well-defined characteristic of the melting transition enables the detection of single-base mismatches between a probe and target DNAs. By measuring the dissociation rates of dsDNA at different temperatures, Reed and Wittwer proposed a method for tracing food products with High Resolution Melting Analysis (Reed and Wittwer 2004).

DNA melting process is usually characterized with the melting temperature. Melting temperature is conventionally defined as the temperature at which the probabilities of finding a given DNA in the hybridized and dissociated states are the same. The melting temperature is closely related to the environment to which the DNA is subjected (Sorokin et al. 1997; Mrevlishvili et al. 1998; Goobes et al. 2003; Khimji et al. 2013; Nakano et al. 2014). Mrevlishvili et al. (Mrevlishvili et al. 1998) found that the DNA melting temperature increases with the increase of the ionic concentration, and the same conclusion has been drawn by Sorokin et al. through a study on the influences of ions on the thermo-stability of dsDNA (Sorokin et al. 1997). Goobes et al. (2003) added crowding reagent PEG into DNA solution, and found that inertia polymers are in favor with the thermostability of DNA. However, the added reagents may also bring opposite effect due to their molecular interactions with the basepairs in dsDNA. Nakano et al. (2014) found that the melting temperature decreases in the presence of PEG, especially in the region with high PEG concentrations. Different reagents may have different effects on DNA melting depending on the competition between entropic effect and correlation effect. Indeed, Khimji et al. (2013) concluded that the melting temperature of duplex DNA is much higher in polyanions than in non-ionic polymers with similar ionic strength due to an additional electrostatic contribution beyond the excluded volume effect.

Apart from these experimental studies, many coarse-grained theoretical models have been developed to address the DNA melting profile (Drukker and Schatz 2000; Knotts et al. 2007; Sambriski et al. 2009a, b; Liu et al. 2010; Ouldridge et al. 2011; Freeman et al. 2011; De Pablo 2011; Liu et al. 2011, 2012). Drukker and Schatz (2000) proposed a beads-spring model which allows for the investigation of hybridization by using simulation. In this model one nucleotide is represented by two sites, and one is for skeleton and the other for base. The model by Ouldridge et al. (2011) is specifically targeted to reproduce the thermodynamics of DNA melting. However, the model cannot account for electrostatics and sequence specificity. Recently, Knotts and De Pablo et al. (Knotts et al. 2007; Sambriski et al. 2009a, b; Freeman et al. 2011; De Pablo 2011) proposed an advanced three-site-per-nucleotide (3SPN) model and its extensions. This model faithfully captures the characteristics of major and minor grooves and the elasticity of DNA, and it involves an ion-concentration dependent long-range electrostatic interaction between the phosphates in two strands. This model is parameterized by fitting the thermal melting experimental data in a constant ionic strength, and unsurprisingly it can well predict the behaviors of DNA chain including melting profile, bubble formations and the mechanical properties of the molecule as a function of ionic concentration. Besides the simulation models, thermodynamic models are also developed for studying the melting process. Very recently, Liu et al. (2010) developed an interesting molecular thermodynamic model from the perspective of phase equilibrium between the association and dissociation states of each basepair. By constructing a proper free energy function which involves the melting enthalpy contribution from each basepair, they showed that the melting curves and melting temperatures of dsDNA in ionic and crowded solutions or in confined spaces could be successfully predicted (Liu et al. 2010, 2011, 2012).

**Fig. 1:**
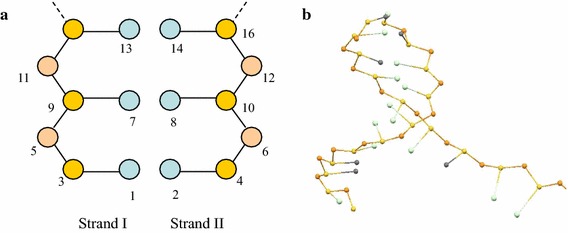
**a** Illustration of 3SPN model for dsDNA: *blue coloured circle* denotes base bead, *yellow coloured circle* for sugar and *orange coloured circle* for phosphate; **b** illustrative process of DNA unzipping

Two different mechanisms on DNA melting have been discussed: slithering and unzipping. It’s widely accepted that long DNA chains display the melting behavior through slithering, while short DNA chains through unzipping (see Fig. 1b for illustration). In addition, insightful investigations on the melting of DNAs with B-form helical geometries have also been reported (Giudice et al. 2001, 2003; Várnai and Lavery 2002; Banavali and MacKerell 2002; Hagan et al. 2003; Giudice and Lavery 2003; Sambriski et al. 2009a, b). By using transition-path sampling, Hagan et al. (2003) have found that the unzipping of dsDNA proceeds via two qualitatively different pathways: in the first one the flipping base breaks its intramolecular hydrogen bonds before it unstacks, and in the other one it ruptures both sets of interactions simultaneously. Almost in all cases, unzipping occurs following a stepwise process in which one base unstacks significantly before the other. The hydrogen bonds break mainly through twisting motions of the bases. Giudice and Lavery (2003) concluded that the base pair lifetimes for canonical B-DNA directly reflect the difficulty of breaking the intramolecular hydrogen bonds and local stacking interactions, while the unusually long lifetimes for A-T pairs within A-tracts reflect the additional constraints imposed by a narrow and rigid minor groove. Sambriski et al. (2009a, b) proposed that in terms of chain length, the free energy barrier of longer oligonucleotides (30 versus 15 base pairs) is higher and slightly narrower, due to increased sharpness associated with the transition. Low ionic strength tends to decrease free energy barriers.

While numerous experimental and theoretical works have been contributed to the determination of melting profile of dsDNA in diverse environments, the melting dynamics of dsDNA, especially for those with short chain lengths, is less understood (Drukker et al. 2001; Banerjee and Pal 2007; Wong and Pettitt 2008; Kenward and Dorfman 2009). Generally, the access of intermediate melting transition state is of great interest. Such information is crucial because it’s helpful to explore the sequence and end effects and to reveal the role of crowding or confinement environment for DNA melting (Perez and Orozco 2010; Toma et al. 2014).

The aim of present work is to study the dynamics of DNA melting in dilute brine environment. The denaturation of short DNA chains is usually employed in Biosensing (Sendroiu et al. 2011; Toma et al. 2014; Loget and Corn 2014) and grafting or anchor technology (Ma et al. 2009; Seela et al. 2011; Zhao et al. 2012; Woller et al. 2012). Besides, it is straight-forward to explore the mechanism of DNA melting by using short DNA chains than using long DNA chains (Wong and Pettitt 2008; Miyoshi and Sugimoto 2008).

## Modelling and simulation details

The 3SPN model developed by Knotts et al. (2007) is employed here to explore the denaturation dynamics of short DNA. The model was developed initially for the investigation of the melting curve. However, as we demonstrate below, it can be utilized to qualitatively explain the melting dynamics. The system under consideration is composed of an isolated dsDNA chain with certain chain sequence. The electrolyte solution is characterized as a continuum with an ion-concentration dependent Debye length scaling the interaction between two charged phosphate beads, i.e.,

1 $$\kappa_{D} = \left( {\frac{{\varepsilon_{0} \varepsilon_{k} RT}}{{2N_{A}^{2} e_{q}^{2} I}}} \right)^{0.5} .$$

Here *κ*_*D*_ is Debye length, and *I* is ionic strength. *ɛ*_0_ and *ɛ*_*k*_ are vacuum permittivity and the water relative dielectric constant at room temperature, respectively. *T* is the absolute temperature of the system and *R* the universal gas constant; *N*_*A*_ is the Avogadro constant and *e*_*q*_ the elementary charge. As demonstrated by Knotts et al. (2007) and Sambriski et al. (2009a, b), such implicit solvent description allows for capturing the ionic concentration effect on melting profile.

The geometry and force field of 3SPN model are detailed elsewhere (Knotts et al. 2007; Sambriski et al. 2009a, b), and here we only give a brief introduction. As shown in Fig. 1a, the 3SPN dsDNA model is composed of two ssDNA strands, and in each strand three kinds of beads are included which represent sugar, phosphate and nucleotide. Each phosphate bead carries a negative elementary charge, and the nucleotide can be Adenine (A), Guanine (G), Cytosine (C) or Thymine (T). For different basepairs, i.e., A-T and G-C, the hydrogen bonding interactions are distinct. At natural state, the geometry of dsDNA can be characterized by the lengths of the connecting bonds and the values of the triangle and dihedral angles. Besides, the backbones of the second chain should be reproduced by simply rotating the first chain along the axis and then shifting up with a given height for a double helix structure. Under perfect matching condition, the structure of a dsDNA is determined once the sequence of nucleotides in either strand is specified, and in this circumstance we conventionally use a sequence composed of A, G, C and T to represent a dsDNA chain.

The interactions involved in 3SPN model include three sets of contributions. The first one is intra-strand interaction accounting for the structural stability of single strand, and the second one accounts for the interaction between two strands involving the electrostatic repulsion between the phosphates in both strands and the basepairing interaction. This baseparing interaction is responsible for the hybridization, and provides a key energy barrier from DNA melting. The last contribution accounts for the elasticity of DNA chain, and specifically the electrostatic interaction among the phosphate beads are included, and the strength of this solvent-mediate interaction is dependent on the ion-concentration in solution. The mathematical formats of those interactions and the involved parameters can be found in the original work (Knotts et al. 2007; Sambriski et al. 2009a, b).

In the present work, four samples of short DNA chains are considered for accessing the melting dynamics. The sequences and numbers of corresponding basepairs (bp) are listed in Table 1.

**Table 1:** Sequences of DNA chains considered in this work

DNA name	Sequence (5′ to 3′)
11 bp	ATC CGT ATG CG
16 bp	ATC CGT ATG CGA TCC G
21 bp	ATC CGT ATG CGA TCC GTA TGC
26 bp	ATC CGT ATG CGA TCC GTA TGC GAT CC

The reasons of choosing above target DNA samples are as follows: firstly, DNA chains of different chain lengths should be considered in order to access the length effect on DNA melting dynamics. Secondly, the sequence effect should be reflected, and thus the other three dsDNA samples are generated by simply repeating, in a whole or half manner, the sequence of the first sample. For simplicity, the four DNA chains are referred to as 11, 16, 21 and 26 bp. The melting temperatures of these dsDNA samples are reported to be within the range of 290–350 K depending on the chain length and the salt concentration (Owczarzy et al. 2004; Freeman et al. 2011).

To implement the force calculation during simulation, we introduce the following method to label the beads in dsDNA chain (see Fig. 1a for illustration): the beads in the first strand are labeled by odd numbers, and the beads in the second strand are labeled by even numbers. In each strand, the beads are labeled from the bottom up and from nucleotide to sugar site.

The Brownian dynamics simulation is carried in NVT ensemble, and the motion of bead *i* is governed by Langevin equation

2 $$m_{i} \frac{{{\text{d}}^{ 2} {\mathbf{r}}_{i} }}{{dt^{2} }} = - \nabla U_{i} - \gamma \frac{{{\text{d}}{\mathbf{r}}_{i} }}{dt} + \varvec{W}_{i} (t),$$

where *m*_*i*_ and **r**_*i*_ are the mass and position, respectively, and *U*_*i*_ is the total potential on bead *i*. The random force *W*_*i*_(*t*) satisfies $\left\langle {\varvec{W}_{i} (t)\varvec{W}_{i} (t^{{\prime }} )} \right\rangle = 6k_{\text{B}} T\gamma \delta_{ij} \delta (t - t^{{\prime }} )$. Here *γ* is the friction coefficient with *γ* = 0.05 ps^−1^. The masses for different kinds of bead are different. The temperature is controlled by Nosé-Hoover chain thermostat (Martyna et al. 1992). The Nosé-Hoover (NH) and Langevin thermostats are combined mainly due to the following consideration: when a base pair is hybridized it is only weakly coupled to the solvent because the bonding interaction dominates in this circumstance. On the contrary, when the base pair is dissociated, the bases are in close contact with the solvent. The main difference between Nose–Hoover-Langevin (NHL) and Nosé-Hoover thermostats is the addition of a Langevin friction and noise terms. This combination has been initiated by Samoletov et al. (2007) for the investigation of DNA replication and transcription, and thereafter applied by many others (Frank and Gottwald 2011; Xu et al. 2014; Zhang et al. 2015).

The initial configuration of the DNA is generated following the method described by Knotts et al. (2007). The differential equation above is solved by using Velocity-verlet method iteratively with a time step of 0.01 ps. The simulation is started with 40,000 steps (0.4 ns) of steepest descent minimization followed by a 1 ns equilibration process during which the system temperature is set as room temperature. After equilibrium, a temperature jump to the final target temperature is carried out (Qamhieh et al. 2009) for studying the melting dynamics. The target temperatures, though high, are chosen to ensure the occurrence of melting within microsecond timescale. Under a given condition, 100 parallel simulations are run, and then the melting time is averaged and analyzed. Similar to the original work (Knotts et al. 2007), when the distance of basepair G-C is larger than 2.8694 Å or that of basepair A-T is larger than 2.9002 Å, and in addition two nucleotide beads move in opposite direction, the basepair is considered as being dissociated. Three factors that may affect the DNA melting dynamics are investigated including temperature, ionic concentration and chain length.

## Results and discussions

### Effect of temperature

**Fig. 2:**
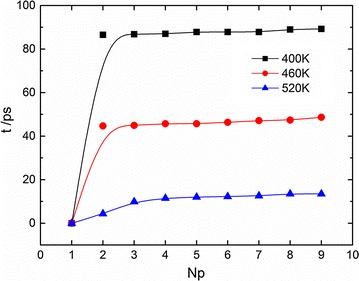
DNA melting time versus the number of dissociated basepairs in 11 bp at different temperatures (0.05 mol/L)

Firstly, the temperature effect on the melting speed is investigated. To characterize this effect, the melting duration time is introduced. The melting duration time is defined as the interval of two time points at which the first and the last basepairs in a dsDNA chain are dissociated. As temperature increases, the average kinetic energy per bead becomes greater, and one would expect that the melting duration time is shorter. Figure 2 plots the melting time in terms of the number of dissociated basepairs in 11 bp at 0.05 mol/L ionic concentration at three temperatures. The melting duration time is determined by time difference between the first dissociation and the last one. For the sake of simplicity, we set the time point of the first dissociation as zero. Note that the first nucleotide in each strand is not paired due to the helix DNA structure, and thus only 9 basepairs within the 11 bp DNA. Three temperatures, i.e., 400, 460 and 520 K, are applied, and these temperatures are much beyond the melting temperature and thus ensure the occurrence of dissociation process. It’s to be noted that although these temperatures are artificial for a physiological system, they enable the physical description and fast simulation studies for the temperature effect. The curves in Fig. 2 confirm that the melting duration time becomes less and less when the temperature increases from 400 to 460 and to 520 K. In addition, we notice that the dissociation of the second basepair generally takes longer time than the subsequent one. However, as temperature increases, such time difference becomes almost neglectable.

**Fig. 3:**
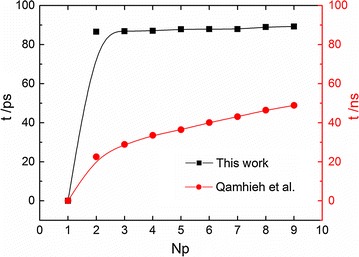
The comparison of DNA melting dynamics at 400 K. The *black curve* *with blocks* is from this work for 11 bp with ionic concentration 0.05 M, and the *red curve with circles* is from (Qamhieh et al. 2009) for a tethered 12 basepairs (A_12_·T_12_) with ionic concentration 0.1 M

Our result generally accords with other simulation findings (Qamhieh et al. 2009; Perez and Orozco 2010). Qamhieh et al. (2009) performed molecular dynamics simulation to study the DNA melting with an atomistic model (Wong and Pettitt 2004), and they explicitly investigated the melting dynamics of 12-basepair (A_12_T_12_) B-DNA duplex tethered to a surface through its 5′-amine linker in the solution of 0.1 M NaCl. For comparison, their simulation data are converted by exchanging the X and Y coordinates and displayed in Fig. 3 (red curve with circles). In particular Qamhieh et al. studied the number of separated basepairs in terms of time and demonstrated that at 400 K the first dissociation of a basepair took much longer time than the other ones, and this generally agrees with our result.

The comparison in Fig. 3 shows that a qualitatively agreement between our simulation and Qamhieh’s result can be found, i.e., the speed of basepair dissociation generally becomes larger and larger during the melting process. One may notice that the time scale of melting duration time in our study is much smaller than that in Qamhieh’s work, and this is very likely due to the difference in modelling, i.e., in Qamhieh’s work the dsDNA chain was described by using an atomistic model while in current work the DNA dissociation process is studied by using a coarse-grained model. Although coarse-grained simulation can give qualitatively or semi-quantitatively similar results as the corresponding all-atom simulation, the simulation time scales in both systems are generally believed to be different. In addition the dsDNA being tethered may present different melting dynamics as in a free space because the tethered dsDNA encounters an extra entropic force from the presence of wall (Zhao et al. 2011). Similar agreement can be obtained by comparing our simulation results to those in Perez’s work. Perez and Orozco (2010) performed a simulation study to gain the real-time atomistic description of DNA unzipping, and they investigated the time evolution of the total number of hydrogen bonds for short dsDNA chains (12-mer) at 368 K, which can be easily converted to the time evolution of total number of separated basepairs. This comparison is very similar to Fig. 3 and thus is omitted here. The agreement with the atomistic simulation results rationalizes our calculation and the analysis on the melting dynamics below.

**Fig. 4:**
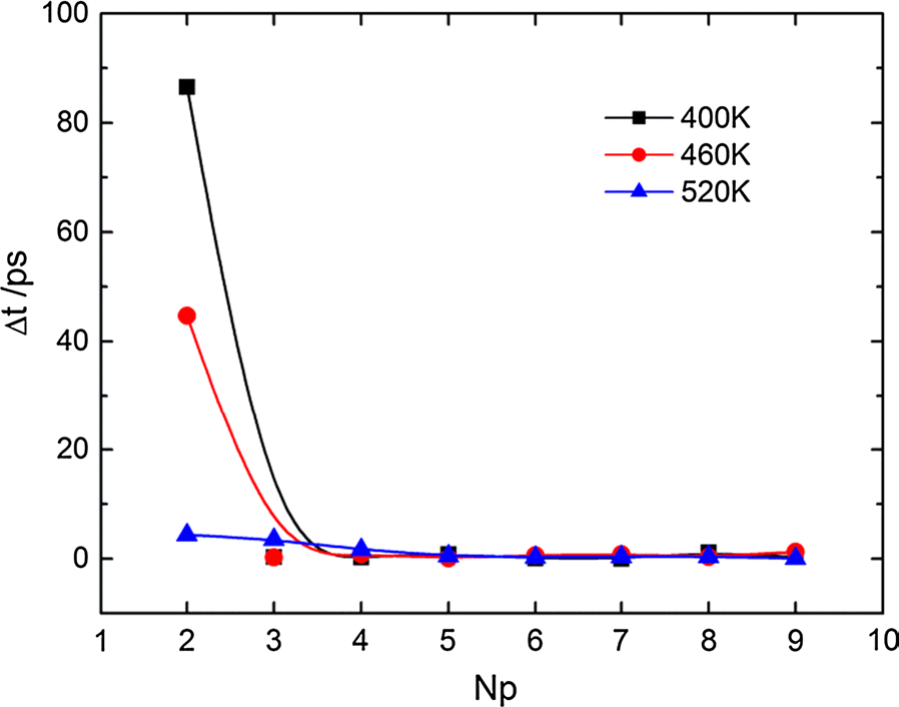
The melting interval time versus the number of dissociated basepairs at different temperatures (0.05 mol/L, 11 bp)

To further analyze the melting dynamics, we plot the time interval between the adjacent dissociations in Fig. 4. It shows that the time interval *Δt* quickly decreases with the increase in the number of separated base-pairs, and becomes almost a constant after the fourth dissociation occurs. This trend indicates that the posterior dissociations are nearly irrelevant with the sequence during the melting process. This is probably because the temperatures considered in current work are much beyond the melting temperature (usually at 350 K), and then the driving force for dissociation dominates while the energy difference in A-T and G-C hydrogen-bonding interactions becomes neglectable. At a relatively low temperature (400 K), the time interval for the dissociation of the second basepair is much longer than the other dissociation after it. Similar trend has been observed during the melting processes of other dsDNA samples.

### Effect of sodium ion concentration

**Fig. 5:**
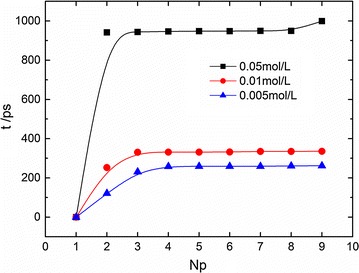
DNA melting time versus the total number of disassociated basepairs (Np) in different ionic concentrations (360 K, 11 bp)

The concentration of salt plays an important role in DNA melting profile (Sorokin et al. 1997; Mrevlishvili et al. 1998), and one can expect the ionic concentration can affect the melting pathway as well. Figure 5 plots the melting time versus the total number of dissociated basepairs in 11 bp at different ionic concentrations. It shows that the DNA melting time becomes shorter in a saline solution with lower ionic concentration, namely a higher melting speed can be achieved by reducing the saline concentration, which elucidates that the stability of DNA can be improved by elevating the ionic concentration, and this trend is likely related to the positive correlation between thermo-stability of dsDNA and ionic concentration as discovered in experiments (Sorokin et al. 1997; Mrevlishvili et al. 1998). Indeed, the addition of ions can screen the electrostatic interaction between phosphate groups in two strands of dsDNA, leading to the improvement of DNA stability. Similar to the temperature effect as discussed in Fig. 2, the difference in dissociation time of each basepair gradually vanishes as the ionic concentration decreases.

**Fig. 6:**
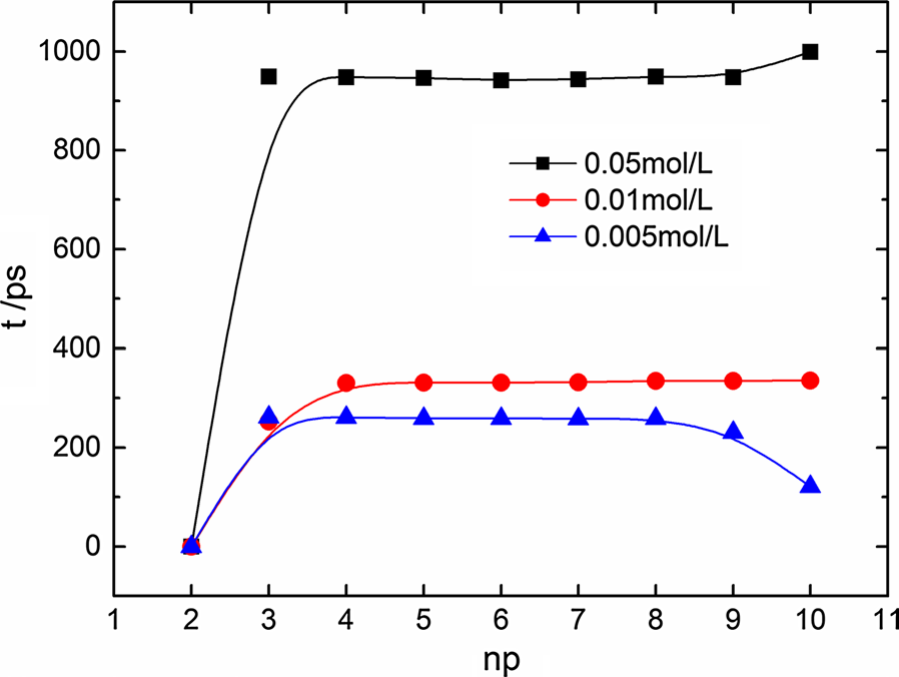
The dissociating time point of the n-th basepair (np) in 11 bp at different ionic concentrations at 360 K

It has been reported that the nature of the transition is controlled by end effects and sequence design (Montrichok et al. 2003). With the help of UV spectroscopy combined with a quenching method, Montrichok demonstrated that the presence of intermediate states could be quantified (Montrichok et al. 2003). By measuring the average fraction of open basepairs and the fraction of completely open molecules, they reported that the symmetric DNA chain (i.e., L42V1, a dsDNA chain with G-C-rich regions at both ends and an A-T-rich region in the middle) in 50 mM ionic concentration opens at both ends during transition, while the asymmetric DNA chain (i.e., L48AS, a dsDNA chain with a G-C-rich region at one end, and an A-T-rich region at the other) opens at one end. Those DNA transitions were characterized during the process of slowly increasing the temperature. The transition details are investigated in our simulation. Figure 6 plots the dissociating time point of the n-th basepair in 11 bp at different ionic concentrations, and it gives the time difference between the dissociations of n-th basepair and the first one and thus reflects the dissociation sequence of the basepairs. We find that the dsDNA opens statistically at one end at high ionic concentration. 11 bp is an asymmetric dsDNA with an A-T-rich region at one end and a G-C-rich region at the other. This melting behavior at high ionic concentration (50 mM) accords with the experimental findings (Montrichok et al. 2003).

While at a low ionic concentration, we find the dsDNA opens at both ends during melting transition. This unusual phenomenon can be explained with the help of interaction energy analysis. As the ionic concentration increases, the Debye length κ_D_ in Eq. (1) decreases, and then the Coulombic repulsion between the phosphates in two strands, i.e., V_qq_, greatly reduces as stated in Table 2. V_qq_, together with the thermal fluctuation force, overcomes the hydrogen bonding attraction for transition. The total driving force that accounts for the dissociation dominates during the melting in our study. When V_qq_ decreases, the driving force decreases, and the net force for DNA transition becomes small. In this circumstance, energy difference on hydrogen-bonding interaction at the G-C-rich end and A-T-rich end becomes significant. This finally leads to a less symmetric probability for opening both ends. Such feature is similar to the reported substrate effect on DNA melting transition, i.e., Qamhieh et al. (2009) found that the melting transition of a tethered dsDNA on a surface was less symmetric than in the solution case where for dsDNA both ends melted with roughly equal probability.

**Table 2:** κ_D_ and V_qq_ in different ionic concentrations (360 K, 11 bp)

Ionic concentration/mol L^−1^	0.005	0.01	0.05
κ_D_/Å	47.203	33.378	14.927
V_qq_/× 10^16^ J	3.772	3.164	1.509

### Effect of chain length

**Fig. 7:**
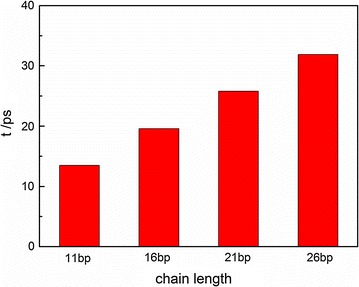
The melting duration time of DNA in different chain lengths (0.05 mol/L, 520 K)

Finally, the chain length effect on the DNA melting dynamics is investigated. Figure 7 plots the DNA melting duration time of different DNA samples at 520 K and at ionic concentration of 0.05 mol/L. As illustrated, the melting duration time is nearly proportional to the chain length. This trend is generally expected. At a high temperature, the melting time interval for each basepair is nearly a constant as discussed in Fig. 4. Because more basepairs in a long DNA chain are to be melted, the melting duration time is supposed to be proportional to the numbers of basepairs. At a low temperature (i.e., 400 K), the melting duration time is less dependent on chain length (not shown here) since the melting duration time at this condition is mainly dominated by the first dissociation regardless of the chain length.

## Conclusions

In the present work, a series of coarse-grained molecular dynamics simulations have been performed for investigating the melting dynamics of short dsDNA chains. By accessing the temperature effect, ionic concentration effect, and chain length and sequence effects, we find that the melting dynamics of short dsDNA chain is no trivial. The addition of ion can improve the stability of DNA. Moreover, the DNA melting has two different pathways: opens at one end, and opens at two ends. At dilute ionic concentration, the dsDNA, regardless of being symmetric or asymmetric, opens at both ends with roughly equal probabilities; while at high ionic concentration, the asymmetric dsDNA chain opens at the A-T-rich end. The comparisons of our simulation results with available data are discussed, and overall good agreements have been found.

The current work reveals the melting dynamics of an isolated short dsDNA chain in saline solution. The majority of DNA in experimental study is long DNA chains and the physiological environment is crowded and geometrically confined. The melting dynamics in practical may be significantly distinct due to the additional steric effect and correlations between DNA chains. Toward that realistic study, a more complex modelling and simulation is required which represents the further direction of this work.
